# Use of Imageless Navigation in the Conversion of Hip Fusion to Total Hip Arthroplasty

**DOI:** 10.7759/cureus.18404

**Published:** 2021-09-30

**Authors:** Paul Kuzyk, Allan Gross, Iain R Lamb, Jeffrey M Muir

**Affiliations:** 1 Department of Orthopaedic Surgery, Mount Sinai Hospital, Toronto, CAN; 2 Clinical Research, Intellijoint Surgical, Kitchener, CAN; 3 Clinical Research, Intellijoint Surgical, Waterloo, CAN

**Keywords:** total hip arthroplasty, hip arthrodesis, hip fusion takedown, computer-assisted navigation, hip conversion

## Abstract

Conversion of hip arthrodesis to total hip arthroplasty is associated with significant challenges, including accurate restoration of leg length and proper orientation of the acetabular component. Computer-assisted navigation provides real-time data on these parameters that may be a useful augment during hip fusion takedown surgery. Here, we present the case of a 64-year-old woman who presented with symptoms related to a left hip arthrodesis. The patient underwent a left-sided hip arthrodesis takedown and conversion to a total hip arthroplasty (THA). Due to the altered anatomical architecture of the fused hip, imageless navigation was used to assist with the conversion to THA. This case demonstrates that in complex hip arthroplasty procedures, where anatomical morphology is altered, navigation technology can be beneficial in addressing the challenges of achieving optimal placement of acetabular components and establishing appropriate leg length and offset.

## Introduction

Hip arthrodesis, i.e., hip fusion, has historically been used in cases of severe trauma or degenerative hip disease [1] and, while successful at minimising joint pain in these severe cases, the drawbacks to hip fusion are obvious. The impediment of normal gait and the cascade of compensatory degenerative changes that accompany it can affect the lumbar spine, knees and contralateral hip, leading to significant disability [2,3]. Thus, there has been a substantial decline in the number of hip arthrodesis procedures performed, and as technology advances, more options for conversion to total hip arthroplasty (THA) are available for arthrodesis patients.

The main benefits of conversion of arthrodesis to THA are the restoration of motion and function to the joint and associated improvements in quality of life [4], with conversion generally indicated for patients experiencing arthrodesis-related low back pain, ipsilateral knee or contralateral hip pain, unfavourable joint alignment or non-fusion [3]. Conversion to THA, however, comes with several challenges. Correcting leg length discrepancy (LLD) and reaming the acetabulum for appropriate acetabular component positioning are particularly complex given the altered architecture of the bone and soft tissue typically observed in arthrodesis [1,3,5]. While careful pre-operative planning may mitigate these concerns, more advanced technologies such as computer-assisted navigation provide more accurate information and may offer a more reliable avenue for improved patient outcomes. The technology we utilise during THA has been demonstrated to be safe and accurate in clinical studies [6,7], as well as in assisting with challenging cases such as Legg-Calve-Perthes disease [8] and complex revision THA [9,10]. As such, surgeons may find benefit in leveraging the ability of navigation to provide accurate, real-time data to improve acetabular and femoral component positioning in arthrodesis conversion. Here, we report a case of hip fusion takedown and conversion to THA performed with the aid of imageless computer-assisted navigation. To our knowledge, this is the first such documented case of its type.

## Case presentation

Patient presentation

A 64-year-old female presented with a chief complaint of right hip, right anterior groin and right thigh pain. She also reported pain in her left knee and left lower back. Relevant history included a left hip arthrodesis following a vehicle accident approximately 40 years prior. Examination revealed that the patient was able to ambulate very short distances with assistance of a single-point cane and used a scooter for travelling longer distances. When ambulating, the patient did so with an antalgic gait. Conservative pain management for the current presentation had included steroid and local anaesthetic injections in the right hip. Physical examination found that the left hip had no range of motion due to arthrodesis, and no apparent strength of the abductors. While LLD was difficult to accurately assess due to a fixed flexion deformation of the left hip, a valgus deformity in the right knee, severe pelvic obliquity and degenerative scoliosis, the left leg was grossly estimated to be 10-15 mm shorter than the right. No neurologic deficit was present.

**Figure 1:**
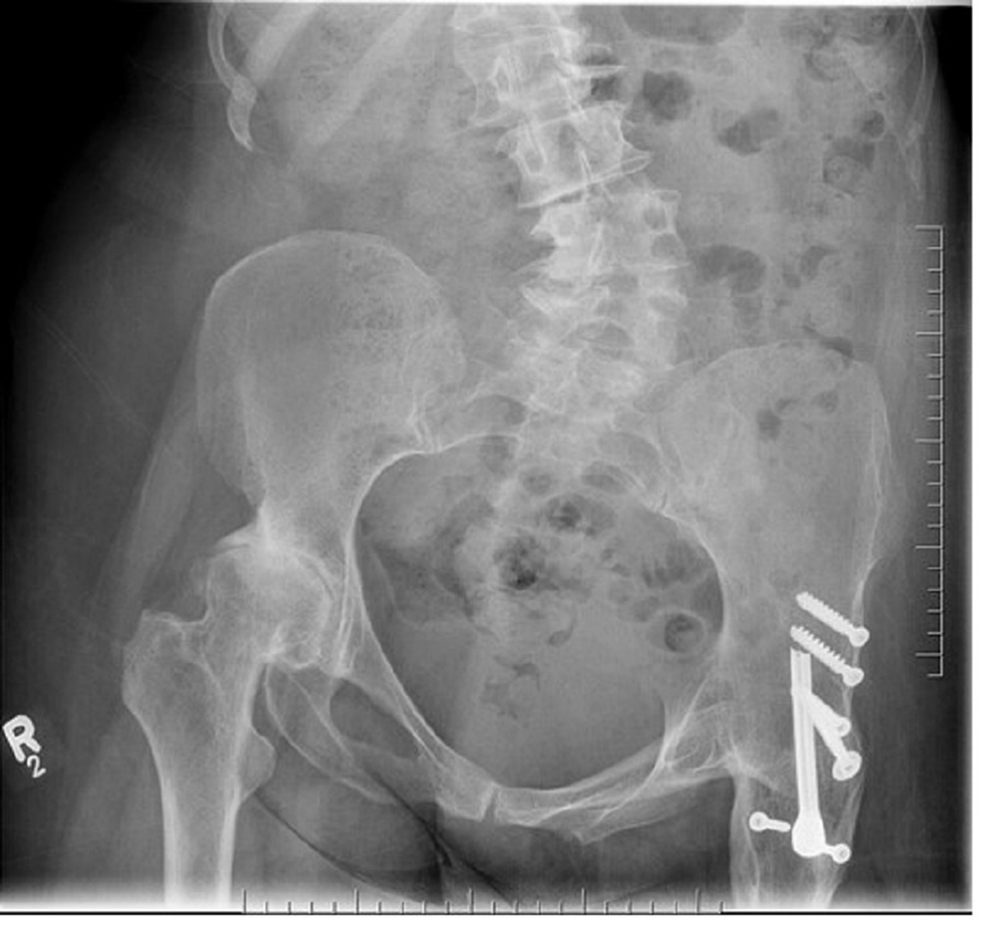
Pre-operative image Pre-operative anteroposterior radiograph of the pelvis, depicting left-sided hip arthrodesis. Advanced degenerative scoliosis of the lumbar spine is also noted.

Pre-operative radiographs (Figure 1) revealed a left-sided hip arthrodesis with the hardware securely in place. Significant pelvic obliquity was also noted on imaging, which further revealed advanced degenerative scoliosis in the lumbar spine and osteoarthritis of the right hip, with a loss of joint space superiorly. While right hip arthroplasty was deemed necessary, it was advised that conversion of the left hip fusion to THA should be completed prior, to optimise the outcome of the right hip THA. The use of computer-assisted navigation was recommended to assist with placement of arthroplasty components.

Surgical procedure

**Figure 2:**
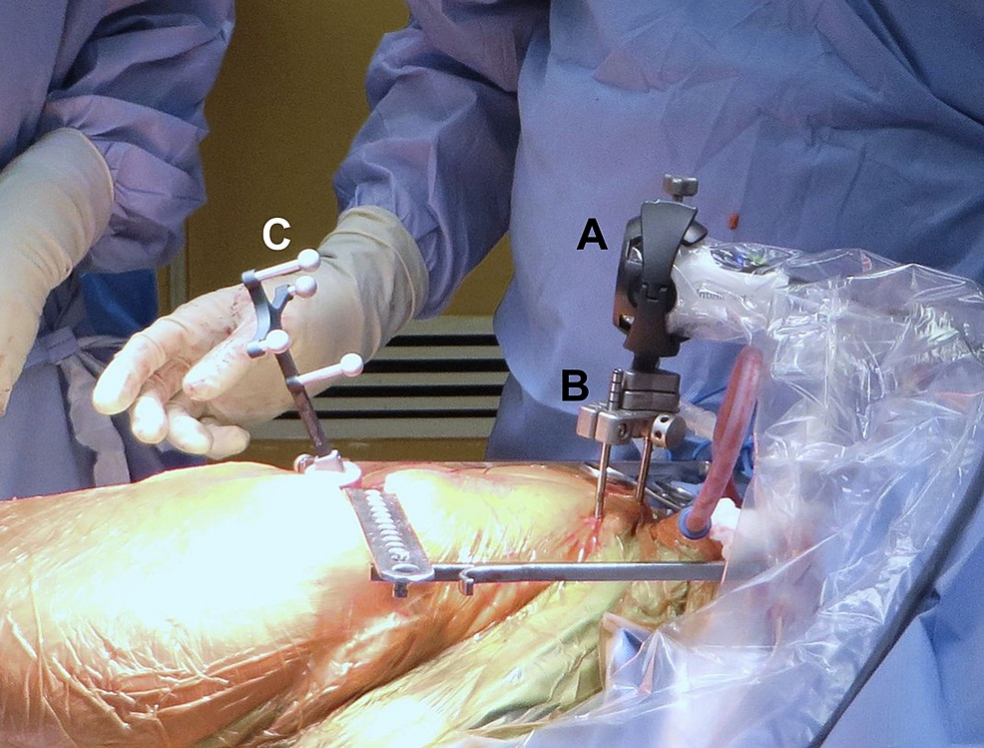
Intellijoint HIP in use The camera (A) sits atop of a pelvic platform (B), which is fixed during surgery to the ipsilateral iliac crest via two bone screws. The camera captures the position and movement of the optical tracker (C), shown here fixed to a platform attached to the greater trochanter to measure changes in leg length and offset. The tracker can also be fixed to instruments (e.g., impactor) to measure the position of the acetabular component.

**Figure 3:**
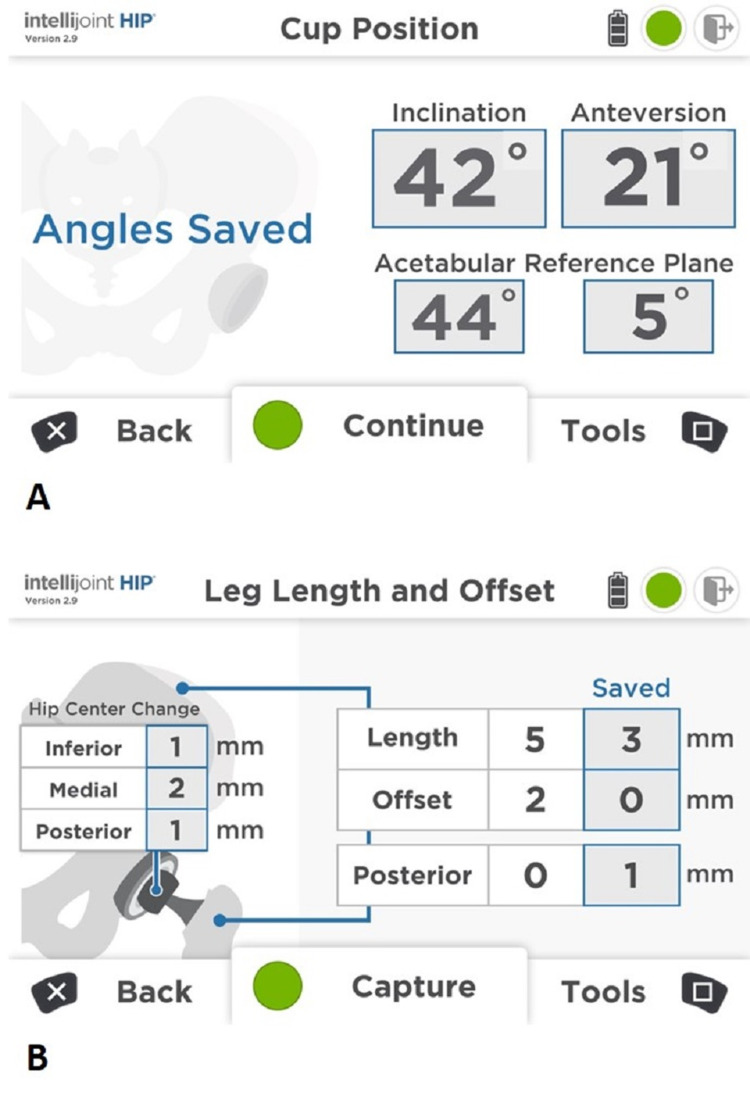
Computer workstation display Measurements of acetabular component orientation (A) and change in leg length and offset (B) are displayed in real time on the computer workstation which sits outside of the sterile field but within view of the surgeon. The workstation is controlled by the surgeon using buttons on the camera or by an assistant using the computer keyboard.

Surgery was performed with the assistance of the Intellijoint HIP® imageless navigation device (Intellijoint Surgical Inc., Kitchener, Ontario, Canada). The patient was placed in the lateral decubitus position, the left hip and lower extremity were prepared and the navigation device was installed as previously described in detail elsewhere [11]. In brief, the device consists of a camera, a computer laptop workstation and an optical tracker. During use, two surgical screws are inserted into the ipsilateral iliac crest via stab incisions and a pelvic platform is fixed to the screws. The navigation camera sits atop the platform, fixed in place magnetically (Figure 2A, 2B). The camera captures the position and movement of the optical trackers (Figure 2C), which can be magnetically attached to a platform fixed to the femur (within the surgical incision) to measure changes in leg length and offset or to surgical instruments (e.g., reamer, cup impactor) to measure reaming angle and acetabular component position. Measurements are displayed on the computer workstation, which sits out of the sterile field but within view of the surgeon, in real time (Figure 3A, 3B). 

Following patient registration and primary incision, the joint was exposed. On inspection, the abductor musculature was atrophied. A trochanteric slide osteotomy was performed via the lateral approach, with previously placed hardware transfixing the joint removed and the femur osteotomized through the fusion mass. The femur was then retracted, and the fused boney mass was reamed to identify the true floor of the acetabulum. Traditionally, we would establish an acetabular target intraoperatively based on identification of the fovea or, in cases where the fovea is not visible, by placing the acetabular component such that its inferior edge is aligned with the base of the ischium. In this case, owing to the altered morphology, these landmarks were not readily visible, necessitating the use of navigation. During acetabular reaming, fixation of the device tracker to the reamer handle allowed for real-time evaluation of reaming angle following each round of reaming. This allowed us to accurately establish a suitable orientation for the new acetabular component consistent with traditional target safe zones. Following preparation of the acetabulum, a 50-mm porous acetabular shell was press fit and stabilised with two screws and a 28-mm constrained liner was then inserted. The orientation of the acetabular component was confirmed with the navigation device as 42° inclination and 10° anteversion by identifying three points on the rim of the shell with a probe/tracker combination, which provided the *in situ* anteversion and inclination measurements.

**Figure 4:**
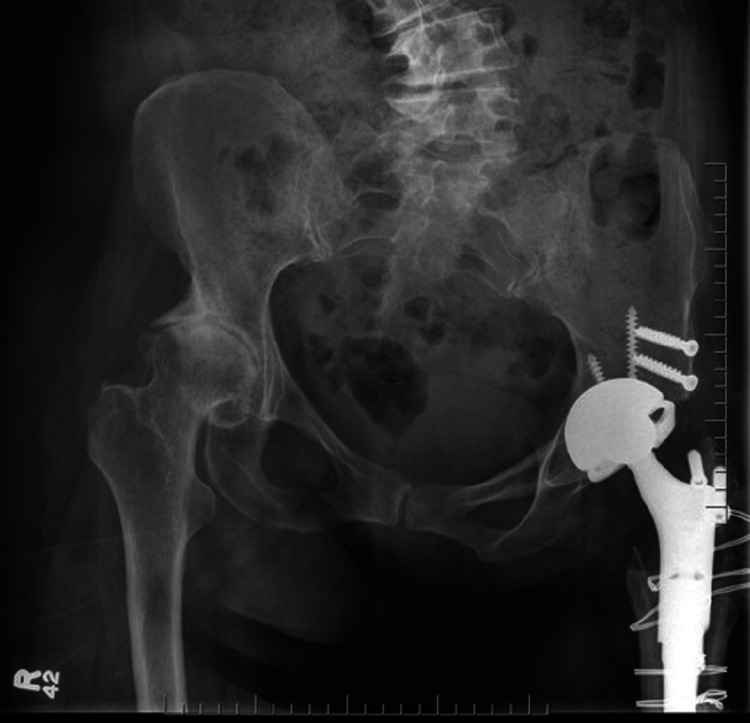
Post-operative image Post-operative anteroposterior radiograph. Conversion of left side hip fusion to THA was successfully completed.

Following completion of the acetabular portion of the procedure, attention was turned to the femur, where a modular, non-cemented femoral component was used. Attachment of the navigation tracker to a platform fixed to the proximal femur provided real-time monitoring of leg length and offset during trialing, allowing for accurate measurement of these parameters before final components were implanted. Given the significantly altered morphology of both the femur and acetabulum, and the lack of traditional landmarks used during femoral trialing, the data provided by the navigation system proved invaluable in accurately establishing a biomechanically suitable leg lengthening. Following femoral broaching and trialing of femoral components and confirmation of desired leg length (+10 mm) and offset (17 mm) changes with the navigation system, final femoral components were implanted and the hip was reduced (Figure 4). 

Total operative time was two hours, with blood loss similar to primary and revision hip arthroplasty (200-300 mL). No transfusion was required. 

Follow-up

**Figure 5:**
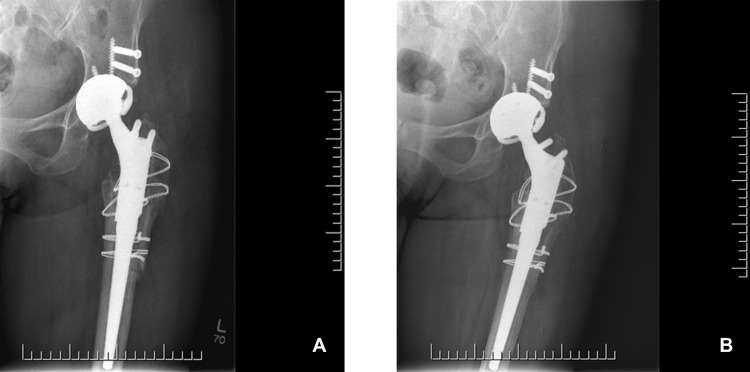
Follow-up radiographs Post-operative radiographs at 7 months (A) and 11 months (B) showed hardware in stable position.

Six weeks post-surgery, the patient was doing well, with no reported complications. She began to bear weight, as tolerated, at three months. Range of motion was greatly improved, with hip flexion measured at 70°, with 10° of internal rotation and 15° of external rotation. The patient was also able to abduct her left hip passively to 20°. Radiographs at seven months post-procedure showed hardware in stable position (Figure 5A). The patient subsequently underwent a primary THA on the right side. At 11 months post-fusion takedown, the patient reported ambulating well, using a single cane as an aid. She reported being pain free, had returned to outdoor leisure activities and was, overall, extremely happy with her progress. Final radiographs at 18 months post-fusion takedown showed hardware of the left hip in stable position (Figure 5B). The patient provided consent for their deidentified information to be used in this case report.

## Discussion

Despite its benefits, the takedown of hip fusion and conversion to THA is associated with complications. The benefits of conversion to THA are many, with recent studies demonstrating increases in Harris Hip Scores of over 20 points [2] and a high level of patient satisfaction and improved quality of life [2,12]. The challenges presented by altered bone and soft-tissue anatomy, however, are substantial, with studies demonstrating a heightened likelihood of instability and loosening associated with arthrodesis conversion, when compared to primary THA procedures [1,2,5]. Chief among the challenges associated with hip fusion conversion are the significant changes in bone and tissue architecture that occur with arthrodesis, which may obscure anatomical landmarks traditionally used in the placement of THA components. The resulting difficulty in properly implanting components can lead to higher rates of acetabular component malpositioning and associated complications. Likewise, the lack of traditional landmarks on the femur increases the difficulty associated with establishing suitable leg length and avoiding a post-operative leg length discrepancy. In our case, these challenges were addressed by the use of computer-assisted navigation, which provided real-time feedback on component orientation and allowed for modification and adjustment prior to final implantation.

The patient in our case had been injured as a young adult and subsequently spent 40 years with essentially no mobility in her left hip. Given that extended period of immobility and the associated boney ingrowth into the fused joint, the ability to adequately prepare the joint for arthroplasty was substantially compromised. The use of navigation demonstrated a two-fold advantage in this case, providing valuable information that would have been otherwise unavailable due to the altered anatomy. During preparation of the acetabulum, the ability to monitor the orientation of the reamer itself in real time allowed us to carefully prepare the acetabulum, allowing us to place the acetabular component in an orientation that would maximise post-operative mobility and stability. By placing the navigation system’s tracker on the reamer handle, we were able to check on its orientation between rounds of reaming, thus providing the ability to make subtle changes during the reaming process. Given the obscured anatomy and lack of traditional landmarks on the ilium, these data allowed for optimal acetabular component placement and minimised the likelihood of malpositioning.

Likewise, we were able to use the navigation tool to closely monitor leg length, again a challenge due to the drastically altered anatomy. During trialing, we were able to assess the reduced joint and resulting leg length following each trial by affixing the tracker to the trochanter. Without a proper femoral head or neck as a reference, and with further alteration due to decades of boney ingrowth around the fused joint, the ability to properly establish an appropriate leg length without the use of navigation would have been very difficult. We were able to lengthen the leg 10 mm in this case which, based on the pre-operative assessment, resulted in a minimal leg length discrepancy. With an arthroplasty procedure on the contralateral side pending, the use of navigation on the arthrodesis side allowed us to prepare the leg such that, following the contralateral THA procedure, the patient would be left with equalised leg lengths and minimal if any leg length discrepancy. This ability to closely monitor leg length and accurately equalise leg lengths has been noted in other conditions where substantial leg length discrepancies of >3 cm exist, e.g., Legg-Calve-Perthes disease [10], although to our knowledge, this is the first documented use of navigation in a hip fusion takedown procedure.

## Conclusions

This report details the case of a 64-year-old patient who presented with a left hip arthrodesis. The integration of navigation and successful conversion to a THA demonstrates that in complex hip arthroplasty procedures, where anatomical morphology is significantly altered, navigation can help address the challenges of achieving placement of acetabular components and establishing appropriate leg length and offset. Eighteen months post-operatively, we confirmed that the components remained stable and in their intended orientation, with the hip restored to a functioning joint, and patient’s quality of life greatly improved.
